# Management of an Extensive Vascular Lesion on the Lip by Photocoagulation with High-Intensity Diode Laser

**DOI:** 10.2174/1874210601711010242

**Published:** 2017-05-16

**Authors:** Luciane H. Azevedo, Dante Migliari

**Affiliations:** 1 Clinician at the General Dentistry Care Center at the Main-Office Building for Social Assistance (SAS), and at the Laboratory of Laser in Dentistry (LELO), University of Sao Paulo, Sao Paulo, Brazil; 2 Department of Stomatology, Division of Oral Medicine Clinic, School of Dentistry, University of Sao Paulo, Sao Paulo, Brazil.

**Keywords:** Vascular malformation, Congenital lip enlargement, Photocoagulation, Diode laser

## Abstract

**Objective:**

Extensive vascular malformations (VM) pose difficulties for an effective management.

**Introduction:**

This article describes a very satisfactory result, both functional and aesthetic, following a management by the technique of photocoagulation using diode laser in an extensive VM lesion involving the lower lip and left buccal mucosa in a 25 year old male.

**Case report:**

The patient reported that the lesion had been present since birth. The whole treatment, carried out under local anesthesia, spread over 6 months since as many as 4 sessions of laser, with a 1.5-month interval in each, were required. The resting period between sessions played an important part in treatment by allowing a time for the recovery of the patient and the shrinking of the lesion.

**Conclusion:**

The patient had no complications during the laser sessions, and his postoperative period was uneventful. No recurrence has been seen after a 2.5-year follow-up.

## INTRODUCTION

Vascular lesions arising at birth mainly include haemangiomas and vascular malformations. Either haemangiomas or vascular malformations can produce a great array of clinical manifestations. Most frequently, vascular lesions cause as small or large swelling on the tissue and show a bluish to purple discoloration on the tissue’s surface. These vascular lesions predominately affect the venous system [1-4], they are of benign nature, and around 50% of them are located on the head and neck region. When located on the face, they can produce a disfiguring aesthetic appearance [1-3].

For a practical diagnosis approach, haemangiomas are usually not present at birth, making their appearance during the neonatal period. Vascular malformations, on the contrary, are often present at birth, and grow in accordance with the individual’s growth and do not tend to regress. Distinction between haemangiomas and malformation is necessary since the former tend to show spontaneous involution over time while the latter has a tendency to persist indefinitely [3-5].

Most (but not all) vascular malformations require treatment. A great challenge arises for management of lesions with deep and extensive involvement of oral tissues.

This article highlights the efficiency of photocoagulation by diode laser for the treatment of an extensive vascular malformation on the lip, yielding a substantial shrinking of the lesion together with a remarkable aesthetic improvement.

## CASE REPORT

A 25-year-old white man was referred to our laser clinic for evaluation and possible treatment of a protuberant and extensive vascular lesion on his lower lip, which had been long bothering him on aesthetic and functional grounds. The lesion was of violaceous color, nearly involving the entire left side of the lower lip, showing a deep infiltration and extension to the buccal mucosa (Figs. **1a** and **b**). On palpation, the lesion was soft and resilient. According to the patient’s history, the lesion had been present since birth. He was first treated with corticosteroids when he was 6 years old, to no avail. This was followed by a surgical intervention, which had provided some reduction of the lesion’s size.

This whole information led to the diagnosis of a vascular malformation. Taking into account the size of the lesion and its potential risk for bleeding, the best therapeutic option for the present case was, under local anesthesia, the use of a high-intensity diode laser in a noninvasive pattern, since it has shown to be effective and safety for the patient as a surgical intervention [6, 7].

### Laser Procedure by Photocoagulation Technique

A high intensity, 3-W diode laser (DMC, São Carlos, Brazil) in a non-contact technique was used. Irradiation was delivered using a flexible quartz fiber of 300 μm in diameter, which was kept 2-3 mm away from the lesion, in continuous wave mode with a mean fluency of 20 J/cm^2^, proceeding with quick circular movements in restricting area of approximately 1 cm^2^, starting on the borders of the lesion. The endpoint of the laser delivering was a sign of blanching on the lesion’s surface (Figs. **1c** and **d**), at which stage the laser was moved to another area. This procedure allows to gradually irradiate the whole lesion. In each session, this procedure was repeated three times with one minute interval between each irradiation to prevent heat damage. Each irradiation session lasted about 15 minutes; the patient’s discomfort was minimal and there was no bleeding. Just after irradiation, the patient developed slight and temporary swelling of the treated area, for which it was necessary to use anti-inflammatory medication. The whole treatment required 4 sessions at intervals of about 1.5 month each (Figs. **1e** and **f**), thus allowing for recovery of the patient and the shrinking of the lesion between the sessions. The last evaluation made after 2.5 years since last irradiation showed that the technique applied on this case had yielded a marked reduction of volume of the lesion along with a very satisfactory cosmetic result (Figs. **1g** and **h**).

## DISCUSSION

Clinical uses of various modalities of laser treatment such as argon laser, neodymium-doped yttrium aluminum garnet (Nd:YAG) laser, carbon dioxide (CO_2_) laser, and diode laser have been found to be safe and effective for the treatment of vascular lesions. Although haemangiomas and vascular malformations are two pathologically separate entities, the therapeutic procedures used for their removal are fundamentally identical [6].

For the purpose of the present case, the high-intensity diode laser was thought to be more appropriate since it is selectively absorbed by hemoglobin. Due to its poor absorption by water, the high-intensity diode laser penetrates into the tissue down to a depth of 4-5 mm. As it passes through the tissue, the laser beam generates heat when absorbed by hemoglobin and thus coagulates tissue (down to a depth of about 7-10 mm) in a process known as photocoagulation. High-intensity diode laser photocoagulation does not generate pigmentary and/or textural changes in treated areas, which are commonly seen when using defocused continuous CO_2_ laser [6-8].

One main advantage of the photocoagulation technique using diode laser is its fast intervention with no bleeding while a patient experiences low or no psychological stress; its drawback, on the other hand, may lie in the requirement that the device should be handled by professional with certified qualification on how to use the equipment with all the required safety measures. Additionally, the cost of the equipment may be a factor for impairing its large use in many oral medicine clinics.

Our patient was 25 years old, had the lesion since birth, and had long been waiting for some effective management in order to have some more joyful in life. The use of photocoagulation technique provided a very successful result on both aesthetic and functional aspects. No recurrence has been observed after a 2.5-year follow-up.

Currently, it appears that the use of powerful laser in photocoagulation technique has gained wide acceptance. It has shown greater advantage over embolization, steroid therapy, cryosurgery, electrodessication, embolization, and intralesional injections of sclerosant agents [6, 7, 9]. Management of powerful laser also comes with a warning that it can generate excessive heat when not used properly, leading to severe thermal damage on the normal tissues around the lesion.

## CONCLUSION

Vascular lesions affecting oral tissues occur with a relative frequency. When they are extensive, such as those accompanying venous malformation, they can be very troublesome to a patient since the treatment for this condition has not been largely available. The photocoagulation technique is an efficient technique and safe for the patient if properly handled. The high-intensity laser using either Nd:YAG or diode is very instrumental for the management either of small or extensive vascular lesions as long as the laser device is managed by experienced professionals

## Figures and Tables

**Figs. (1a and b):**
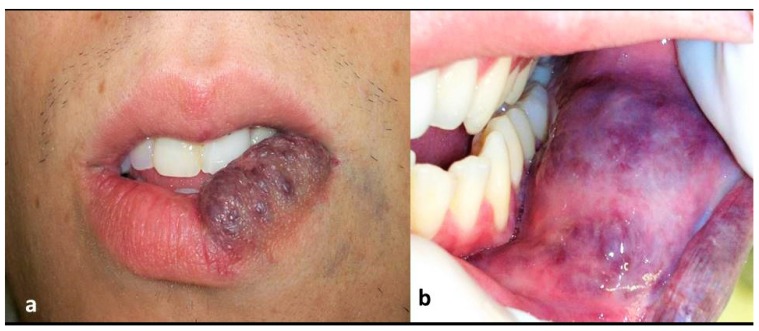
A bluish and extensive nodular-like lesion involving the left side of the lower lip and with a large involvement of the buccal mucosa.

**Figs. (1c and d):**
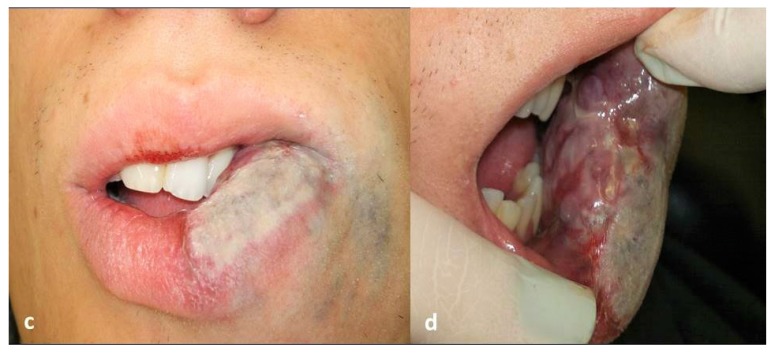
The blanching on the lesion’s surface during laser irradiation; this being a warning sign to stop the irradiation procedure on that area.

**Figs. (1e and f):**
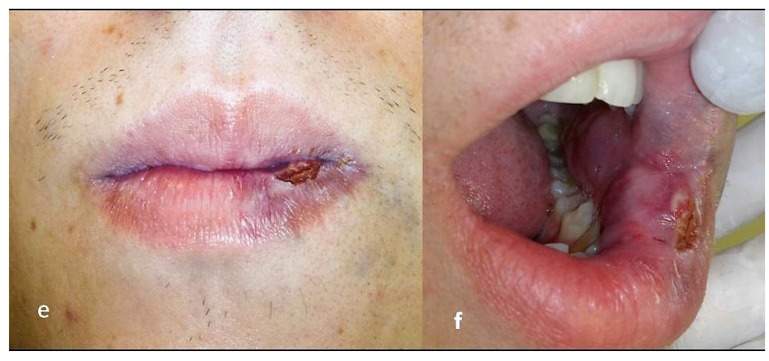
Both pictures show a reduction of the size of the lesion after 45 days since the first irradiation with diode laser. It is possible to notice the healing crust on the lip.

**Figs. (1g and h):**
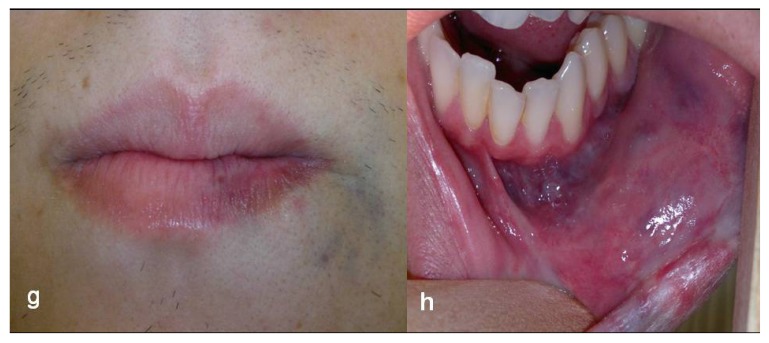
Photographs taken 2.5 years after last session of laser irradiation show a remarkable reduction of the size of lesion along with a very satisfactory cosmetic result; only a discreet bluish area is observed around the corner of the mouth, as the patient is able to close his lips properly.
